# Prevalence of Hepatitis B in Insular Regions of Southeast China: A Community-Based Study

**DOI:** 10.1371/journal.pone.0056444

**Published:** 2013-02-20

**Authors:** Ping Chen, Chengbo Yu, Bing Ruan, Shigui Yang, Jingjing Ren, Weijian Xu, Zhuanbo Luo, Lanjuan Li

**Affiliations:** 1 The State Key Laboratory for Diagnosis and Treatment of Infectious Diseases, The First Affiliated Hospital, School of Medicine, Zhejiang University, The Key Laboratory of Infectious Diseases, Zhejiang Province, Hangzhou, China; 2 School of Medicine, Zhejiang University, Hangzhou, China; Drexel University College of Medicine, United States of America

## Abstract

**Objective:**

Hepatitis B virus (HBV) infection remains a significant public health problem. The purpose of this study was to investigate the seroepidemiology of HBV in people living in the insular regions, and to provide the most recent baseline data for planning and monitoring of health.

**Methods:**

A cross-sectional, community-based survey was conducted without age restriction, on two isolated islands, Zhoushan and Yuhuan, China. The study sample was selected by random multistage cluster sampling. Serological samples and demographic information were collected from 15878 participants.

**Results:**

The prevalences of anti-HBV core antibody (anti-HBc), hepatitis B virus surface antigen (HBsAg), and anti-HBV surface antibody (anti-HBs) were 33.1, 10.4, and 56.1%, respectively. We found statistically significant differences of HBV markers in men versus women (*P*<0.01). The prevalence of HBV infection increased with age. There were significant differences in the rates of HBsAg and anti-HBc positivity between the two islands (*P*<0.01). Alanine aminotransferase (ALT) levels were elevated (>38 IU/L) in 15.6% and 7.2% of the HBsAg-positive and negative groups, respectively. Elevated ALT levels were significantly higher in males (12.0%) compared with females (5.8%) (*P*<0.01). The α-fetoprotein (AFP) positivity rate was 0.6% in HBsAg-positive participants over the age of 30.

**Conclusion:**

Due to the geographic location, we found that the HBV prevalence and potential for the development of hepatocellular carcinoma remained high in insular regions of southeast China, and are far above the national figures. Although a vaccination program has been in effect over the last 20 years, several additional measures should be adopted by the government to limit the spread of hepatitis B. These include the management of high risk persons and the floating population living on the islands, expansion of the immune population, and increased health education for fisherman.

## Introduction

HBV infection remains a severe public health problem worldwide [1], [2]. Each year around the world, HBV infection is responsible for about one million deaths due to liver failure, cirrhosis and more than 75% of the hepatocellular carcinomas world-wide develop from HBV infection [3], [4].

Prior to 2006, China had been classified as an area with high prevalence of HBV [5]. According to a national study published in the early 1979, the overall prevalence of chronic HBV carriage was approximately 8.75% [6], which rose to 9.75% in 1992 [7]. Following recommendations from the WHO, the Expanded Program on Immunization (EPI) for infants was established in China in 1992, and caused a significant decline in HBV prevalence to 7.18% according to a national survey in 2006 [8]. Moreover, significant variations in the seroepidemiology of HBV across regions in China were found, with the highest rate observed in the mid-south, and lowest rate in northern China. The prevalence of HBsAg in Zhejiang Province, which is located in the south of China, was found to be over 11% in 1992, higher than the national rate, but had declined to 5.8% by 2001 [9].

In the insular regions of Zhejiang Province, due in part to a more developed economy, and a high floating population, the prevalence of HBsAg in the residents was found to be 19.33% in 1997, notably higher than that in Zhejiang Province as a whole [10]. Although the EPI, which started from 1992 in these islands, was considered a success in this region, an epidemiological study of 3657 randomly selected fishermen in the coastal islands of Zhejiang Province found that the prevalence of anti-HBc, a marker of HBV exposure was 61.4%, and the prevalence of HBsAg, a marker of active infection, was 15.5% in 2007 [11]. This prevalence rate, although slightly lower than in the past, is still much higher than that for the entire Zhejiang Province (6.75%) [12].

However, to date, there have been very few HBV seroepidemiological studies on isolated islands. The community positivity rate of HBsAg in this region, and related risk factors remain unknown. The purpose of this project was to conduct a community-based epidemiological study to determine the prevalence of HBV infection in two major islands of southeast China, and to provide the most recent baseline data for planning and monitoring of health.

## Methods

### Study Population and Sampling Strategy

With the support of the Mega-Project for National Science and Technology Development for the “11th Five-Year Plan of China”, and the Department of Health of Zhejiang Province, we conducted an investigation on two major islands of Zhejiang Province from October 2010 to March 2012. Two coastal municipalities were randomly chosen. From each municipality, we selected one insular-county/city (equivalent to a county). Zhoushan and Yuhuan are two isolated islands which are connected to the mainland by a bridge. Most of the people make a living by fishing. The towns/subdistricts in each region were divided into three levels according to economic development. In each level, one town/subdistrict was randomly selected. Finally, within each town/subdistrict, three villages/communities were also selected according to the economic level. A list of residents from the resident’s committee was obtained using a registration book.

After receiving appropriate training by the lead researchers for this study, the physicians of each participating hospital began to conduct medical examinations, interviews, and laboratory tests on subjects who volunteered for free medical and health examinations. Altogether, approximately 360 physicians from 18 hospitals in the two insular counties were invited by the Department of Health of Zhejiang Province to participate. All local residents and migrant workers who had lived on these islands for >3 months (*i.e.*, moved to the area before July 1, 2010) were screened for inclusion. The final average participation rate was about 60% among all the participating sites. The information collected consisted of demographics (sex, age, occupation, nationality, and marital status), residency status, medical history (e.g., blood transfusion, hepatitis B, allergies), and laboratory tests (HBsAg, anti-HBs, anti-HBc, AFP, ALT assays, and liver ultrasound (US)). Patients who had been diagnosed with HCV, HDV, HIV, immunosuppression disorders, or were on antiviral therapy, had alcohol abuse, or other liver diseases were excluded from the study.

All participants provided informed written consent and the informed written consent from the next of kin on the behalf of the minors/children participants. The data were analyzed anonymously. The study was approved by the Ethics Committee of The First Affiliated Hospital at the School of Medicine of Zhejiang University.

### Serological Testing

A 5 mL venous blood sample was collected from each participant using strict hygiene and safety guidelines. The patient demographic information, including name, sex, age, and district, were recorded. Blood samples were kept in a low temperature container (controlled from 4–10°C) and delivered to Adicon Clinical Laboratories (Hangzhou, China) on the same day for sample processing and serological testing.

Commercially available enzyme immunoassay kits (Acon Biotech Co., Hangzhou, China) were used for HBsAg, HBsAb, anti-HBc measurements. Verification of elevated test results was carried out by retesting the samples using the same kits. Only samples that were positive on both tests were considered to be true positives. For the purpose of analysis, HBsAg positivity was considered indicative of current HBV infection. Anti-HBc was used as an indicator of previous exposure to HBV. Isolated HBsAb positivity was taken to indicate successful HBV vaccination. Individuals who tested negative for all HBV serological markers were considered to be non-immune and non-infected.

### Alanine Aminotransferase Levels and Hepatocellular Carcinoma Screening

ALT is the most commonly used enzyme in the evaluation of liver disease. Serum ALT quantitation was performed within 2 hr of arrival at the laboratories. Quantitation was achieved using a coupled enzyme and indicator reaction that utilizes pyruvate for a kinetic determination of NADH consumption measured by an Architect C8000 automated biochemical analyzer (Abbott Laboratories, Abbott Park, IL, USA). Abnormal ALT values were defined as greater than 38 IU/L, representing 2.25 SD above the mean for normal subjects. Verification of elevated test results was carried out by retesting the samples using the same kits. Only samples that were positive on both tests were considered to be true positives. HCC community-based preliminary screening was performed for HBsAg-positive subjects by detection of α-fetoprotein (AFP) and imaging of the liver by ultrasound (US). Qualitative α-fetoprotein (AFP) screening for HCC was performed on participants >30 years old (yo) who were HBsAg-positive, using an AFP EIA Test Kit (BoSai, Zhengzhou, China). Liver US was conducted by the doctors from the radiology department of each community health center. A preliminary diagnosis of HCC was made based on the presence of a positive AFP and a focal mass consistent with a tumor detected by US.

### Data Analysis

Data were managed and analyzed using SPSS software version 17.0 (SPSS, Inc., Chicago, IL, USA). For categorical variables, we used the chi-square test to determine group differences. A *P*<0.01 was considered to be statistically significant. The HBsAg, anti-HBs and anti-HBc carrier rates were standardized by age and sex according to the 2010 Zhejiang population data [13] ^.^ The 95% confidence intervals (CI) for the proportion of individuals who were HBsAg-positive, stratified by age and sex, as well as for the whole group, were calculated.

## Results

### Characteristics of the Study Group

A total of 15878 serum samples were tested. Information on age or sex was missing for 881 participants (5.5%). Thus, a total of 14997 samples were analyzed. To allow comparisons without distortion by age and sex, the prevalence was directly standardized to the age and sex distribution of all participants in the study population. In this study, the age groups and proportion of each were: <20 yo (29.34%), 20–29 yo (14.46%), 30–39 yo (20.08%), 40–49 yo (13.94%), 50–59 yo (10.38%), 60–69 yo (6.83%), and ≥70 yo (4.97%) with the same sex proportion [13]. Demographic characteristics of the study population are shown in Table 1.

**Table 1:** Demographic data of the study population.

		Participants	Number	Male	Mean
Region	Population	interviewed	valid	percent (%)	age±SD (years)
Zhoushan	1121300	8932	8346	33.80	55.6±13.6
Yuhuan	411000	6946	6651	44.60	41.1±21.4
Total	1532300	15878	14997	38.60	49.2±18.9

### Prevalence of HBV by Age and Sex

**Figure 1:**
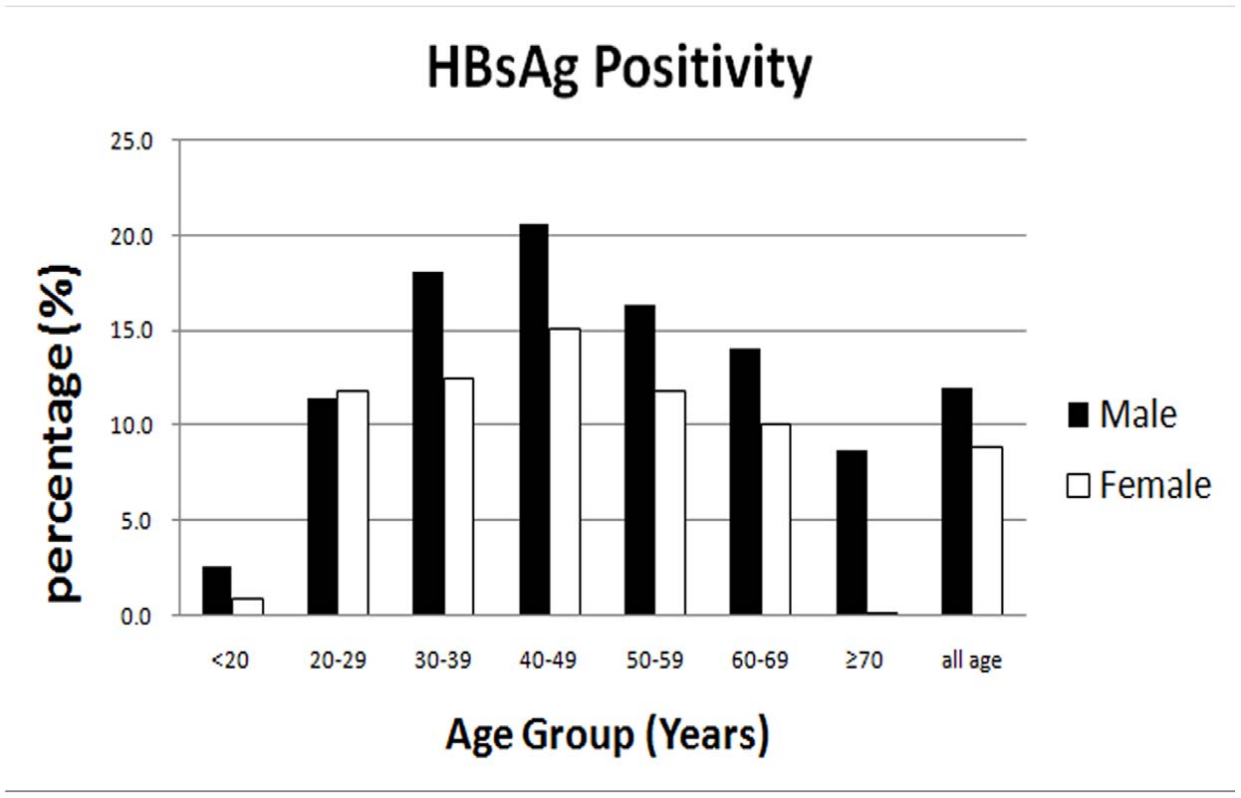
Age-specific prevalence of hepatitis B virus surface antigen (HBsAg).

**Figure 2:**
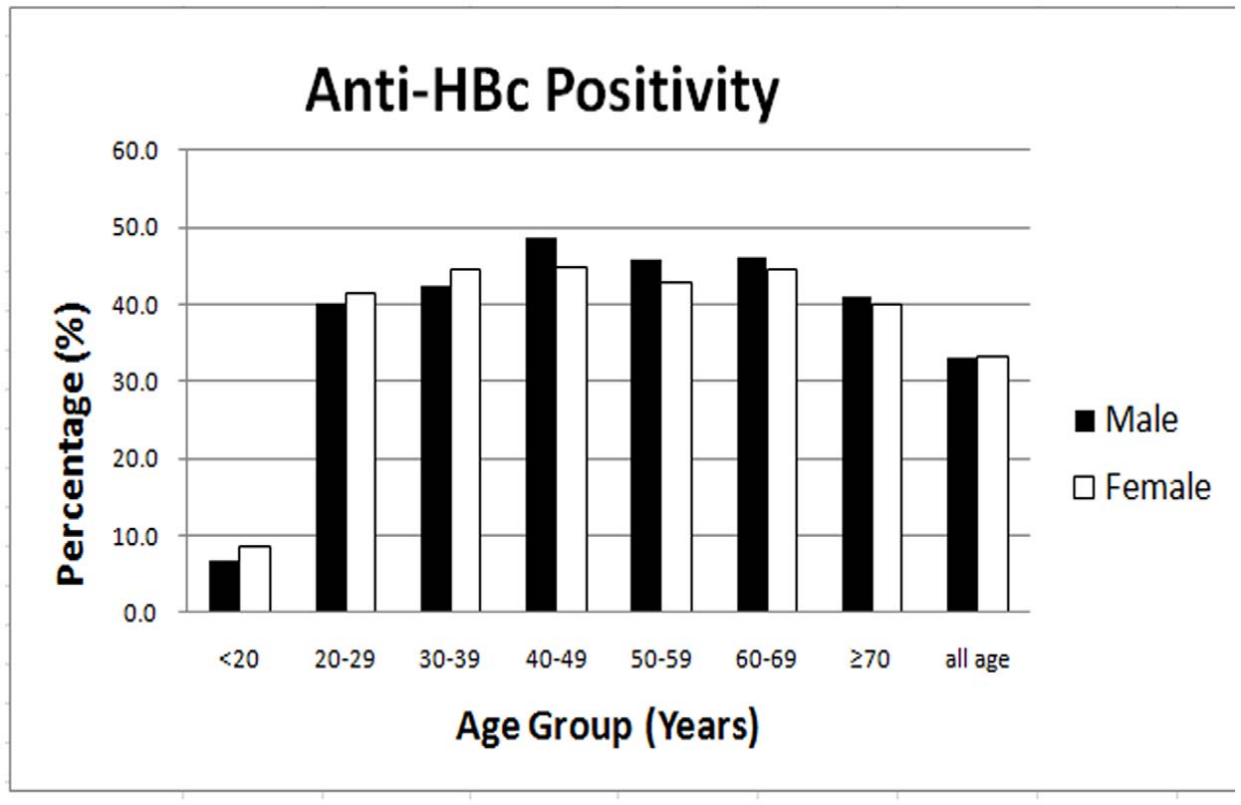
Age-specific prevalence of anti-HBV core antibody (anti-HBc).

A total of 1763 individuals tested positive for HBsAg (Table 2). The prevalence of HBsAg positivity, a marker of current infection, in this population was 10.4% (95% CI: 9.9–10.9). The infection rate increased gradually from 1.7% in those <20 yo to 17.0% in those 40–49 yo, reaching a peak in this decade of life, and declining thereafter (Table 2). The prevalence among male participants (12.0%; 95% CI: 11.2–12.8) was significantly higher (*P*<0.01) compared with that among female participants (8.8%; 95% CI: 8.3–9.3) (Figure 1).The prevalence of anti-HBc, a marker of HBV exposure, was 33.1% (95% CI: 32.4–33.8) overall, but did not vary significantly between the genders. The study showed that exposure increased with age both in males and in females, and reached a peak in the 40–49 yo group, declining thereafter, indicating exposure to HBV had occurred in all age groups (Figure 2).The prevalence of anti-HBs increased from 50.9% to 57.7% in the 20–69 yo group, and more than 60% among those ≥70 yo. Isolated anti-HBs, evidence of HBV vaccine-induced immunity, was found in approximately 39.3% (95% CI: 38.5–40.1) (Table 2).

**Table 2:** Prevalence of hepatitis B virus markers, stratified by age group.

	Only anti-HBs		HBsAg		anti-HBc		anti-HBs	
	No.positive/		No.positive/		No.positive/		No.positive/	
	no. tested	%	no. tested	%	no. tested	%	no. tested	%
<20	943/1725	54.7	30/1725	1.7	132/1725	7.7	1019/1725	59.1
20–29	144/550	26.2	64/550	11.6	225/550	40.9	280/550	50.9
30–39	450/1439	31.3	206/1439	14.3	630/1439	43.8	777/1439	54.0
40–49	1038/3083	33.7	525/3083	17.0	1420/3083	46.1	1676/3083	54.4
50–59	1324/3637	36.4	487/3637	13.4	1596/3637	43.9	2099/3637	57.7
60–69	947/2648	35.8	307/2648	11.6	1194/2648	45.1	1504/2648	56.8
≥70	774/1915	40.4	144/1915	7.5	775/1915	40.5	1191/1915	62.2
Total	5620/14997	39.3	1763/14997	10.4	5972/14997	33.1	8546/14997	56.1

The proportion of individuals who were positive were standardized by age.

### Prevalence of HBV Stratified by Participant Islands

In Table 3, HBV prevalence was calculated according to participant gender in each district. The total prevalence was calculated by adjusting for the population of each island in addition to sex ratio. The prevalence of HBsAg was significantly higher (*P*<0.01) in the Zhoushan group (14.8%; 95% CI: 14.0–15.6) than in the Yuhuan group (9.1%; 95% CI: 8.4–9.8). On each island, the prevalence of HBsAg was higher in males than in females. The prevalence among male individuals in Zhoushan was 17.1% (95% CI: 15.7–18.5).The prevalence of anti-HBc was 43.3% and 35.7% in the Zhoushan and Yuhuan groups, respectively. The prevalence of anti-HBc in the Zhoushan group was significantly higher than in the Yuhuan group (*P*<0.01), whereas there was no significant difference between males and females on either island. The prevalence of anti-HBs showed a trend similar to the prevalence of HBsAg in the two island groups, with prevalences of 59.1% and 54.8% in the Zhoushan and Yuhuan groups, respectively. The prevalence of vaccination-induced immunity (isolated anti-HBs) was also higher (*P*<0.01) in the Zhoushan group (Table 3).

**Table 3:** Prevalence of HBV markers, stratified by region.

		Only anti-HBs			HBsAg		anti-HBc		anti-HBs	
		No.positive/			No.positive/		No.positive/		No.positive/	
Region		no. test	%		no. test	%	no. test	%	no. test	%
Zhoushan	Male	1149/2818	40.8		481/2818	17.1	1228/2818	43.6	1675/2818	59.4
	Female	2066/5528		37.4	697/5528	12.4	2371/5528	42.9	3224/5528	58.3
	Total	3215/8346		39.1	1178/8346	14.8	3599/8346	43.3	4899/8346	59.1
Yuhuan	Male	1110/2966		37.4	300/2966	10.1	1043/2966	35.2	1617/2966	54.5
	Female	2405/3685		35.1	295/3685	8.0	1330/3685	36.1	2030/3685	55.1
	Total	55.1/6651		36.3	595/6651	9.1	2373/6651	35.7	3647/6651	54.8

The proportion of individuals who were positive were standardized by gender.

### Hepatocellular Carcinoma Screening in the HBsAg Positive Group and ALT Levels

**Figure 3:**
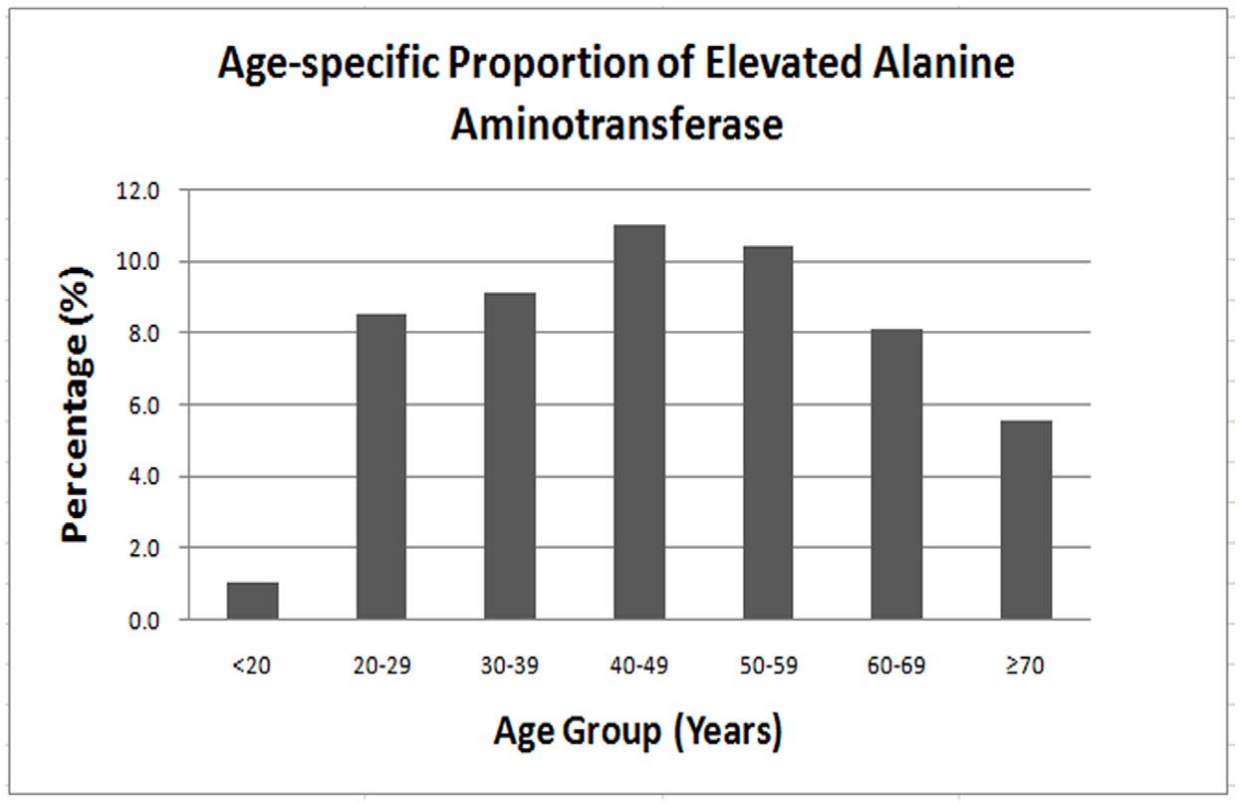
Age-specific proportion of elevated alanine aminotransferase.

There were ten HBsAg-positive patients, three female and seven male, >30 yo (0.6%) who were AFP positive. However, no tumors were found by liver US. The mean (±SD) ALT was 21.1 (±19.0), and the proportion of participants with elevated ALT (≥38 IU/L) was 8.2%. The HBsAg-positive group had a significantly higher level of ALT (27.7 IU/L) compared with the HBsAg-negative group (21.0 IU/L). The proportion of patients with elevated ALT was significantly higher in the HBsAg-positive group (15.6%) compared with the HBsAg-negative group (7.2%)(P<0.01). In both HBsAg-positive and HBsAg-negative groups, the proportion with elevated ALT was significantly higher in male than in female participants (*P*<0.01) (Table 4). In all participants, the proportion of elevated ALT was markedly higher in males (12.0%) than in females (5.8%)(P<0.01). The male participant average ALT level (23.2 IU/L) was higher than that of the female participants (18.3 IU/L). The proportion of patients with elevated ALT significantly decreased with age, from 10.7% in the 40–49 yo group to 5.5% in the 70 yo group (*P*<0.01) (Figure 3).

**Table 4:** Number and proportion of elevated ALT in HBsAg positive and HBsAg negative groups.

	HBsAg-positive						HBsAg-negative					
	<38IU/L			>38 IU/L			<38 IU/L			>38 IU/L		
	No.positive/	Mean		No.positive/	Mean		No. positive/	Mean		No.positive/	Mean	
Serum		ALT±SD			ALT±SD			ALT±SD			ALT±SD	
ALT	no. test	(IU/L)	%	no. test	(IU/L)	%	no. test	(IU/L)	%	no. test	(IU/L)	%
Male	588/761	26.8	77.3	173/761	74.5	22.7	4500/5023	17.2	89.6	523/5023	60.4	10.4
		±27.3			±74.6			±7.4			±30.2	
Female	892/992	18.4	89.9	100/992	77.8	10.1	7791/8221	15.5	4.89	430/8221	55.4	5.2
		±7.1			±79.0			±6.7			±24.4	
Total	1480/1753	22.0	84.4	273/1753	75.7	15.6	12291/13244	16.1	92.8	953/13244	58.2	7.2
		±19.0			±76.1			±7.0			±27.8	

## Discussion

In Zhejiang Province, the EPI vaccination program is considered a success. Since program initiation in 1992, the high risk hepatitis B designation has moved from infants and children (0–9 yo) and adults (30–39 yo) to middle aged adults (40–49 yo) [14]. In the current study, the infection rate of insular residents 40–49 yo was the highest, which is consistent with the findings of a previous epidemiological study. It appears that government programs should continue to target island persons, particularly those in the 40–49 yo group, for HBV-related education, and screening to reduce HBV spread among a wider population.

The national HBsAg prevalence in individuals aged 1–59 yo has been reported to be 7.18% in 2006, which represents a 26.3% decrease from 9.75% in 1992. The rate of HBsAg positivity in insular regions of southeast China fell from 19.93% in 1997 to 15.5% in 2007, a reduction of 22.2%, which is similar to the trend in the rest of China. This may be related to the efficient implementation of the vaccination policy in China. Data obtained from the national survey in 2005 revealed that the HBV vaccination series completion rate of infants was approximately 90.7% in rural areas, and 95.0% in urban areas [15]. The completion rate in Zhejiang province reached 93.03% by 1997, and the coverage rate has increased over the years [16].

The rate of HBsAg positivity varies considerably in different regions of China. For example, the rate of HBsAg positivity in Guangdong Province is 12.45% [17], but only 3.49% in Beijing [18]. Our study demonstrated that HBV infection is still highly endemic in insular regions where 33.1% of people have been exposed to HBV, with about 10.4% having markers consistent with chronic HBV infection. Although the prevalence in insular regions has declined compared with that in 2007, when the exposure rate was 61.4% and the HBsAg positivity was 15.5% [11], it still much higher than the latest result of 6.13% in Zhejiang Province as a whole [19]. Zhejiang Province is an affluent coastal province. Because of the port economy, the insular regions have a more developed economy. However, the high floating population and the special lifestyle on the islands may negatively influence the prevalence of HBsAg. At present, the vaccination program is only for newborns on these islands. According to the current study, the government should consider investing more funds for vaccination of individuals in the insular regions, especially for the high risk group>20 yo who lack protective antibodies, and who were not vaccinated since 1992. Also, the government should pay more attention to the floating population compared with the other districts of China.

The prevalence rate of anti-HBc on the islands increased significantly with age, as other studies have observed[20]–[22]. This indicates that horizontal transmission still plays an important role in China. In 20–29 yo individuals, up to 40% of people living on the islands have already been exposed to the HBV, confirming that HBV infection occurs at a high rate in early life.

The prevalence of HBsAg positivity was found to be significantly associated with gender (males: 21.9% vs. females: 14.7%; *P*<0.01) in the current study, which is consistent with previous observations[22]–[24]. This finding may reflect pathogenesis-related mechanisms involving sex hormones. However, it is also possible that the findings are related to differences in lifestyle or behavior between males and females, such as smoking, drinking, extensive social range, physical activity, dining out, hygiene, and sexual activity. Particularly in insular regions, the activity of fishing is male dominated. The fishermen spend a lot of time eating and living together at sea each year. They do not pay attention to hygiene and there are different lifestyles and diets compared to non-fishermen. Therefore, health education urgently needs to be adjusted to the fisherman's lifestyle.

The study also compared the difference between the Zhoushan and Yuhuan groups, and showed that the positivity rate of HBsAg and anti-HBc in the Zhoushan group was higher than those of the Yuhuan group. Zhoushan is located in the north part of Zhejiang Province with a more developed economy and prosperous ports than Yuhuan. Moreover, the large floating population in Zhoushan increases the difficulty of health education and management [25]. This indicates that in different insular regions, at least in part because of the differences in economic development and the floating population, the prevalence of hepatitis B is clearly different. Both these two areas were included in the EPI in 1992, whereas Zhoushan initiated immunization of children in the floating population in 1999 [26]. Yuhuan instituted immunization of children in 1992. This may explain the observed difference in anti-HBs prevalence between the two islands in the current study.

ALT levels were found to be significantly related to HBsAg status on the islands. The HBsAg-positive group had a higher level of elevated ALT compared with the HBsAg-negative group (15.6% vs. 7.2%; p<0.01). Abnormal ALT levels were significantly more frequent among HBsAg carriers than among those who were not.

The association between ALT and age or gender was also found in the current study, which agrees with previous studies which reported that males were associated with higher levels of ALT than females. However, unlike previous studies, we found that individuals ≥50 years did not have higher levels of ALT [27], [28].

A population-based cohort study from Japan found that even virus-negative participants with an ALT level exceeding 30 IU/L had a statistically higher risk of HCC than virus-positive patients with an ALT level under 30 IU/L [29]. The latter comprised a large proportion of participants in the current study. However, most of them were asymptomatic. Therefore, it is important for patients to participate in periodic examinations by the government.

This is the first study in China to screen for hepatic tumors in isolated islands on the basis of AFP and liver US in the general community. Although no cases of HCC were identified, we found the prevalence of abnormal AFP among HBsAg-positive individuals aged >30 yo was 0.6%, far above the recently reported 0.2% found in Zhejiang Province as a whole [14]. Hepatocellular carcinoma is the fifth leading malignancy and the third most deadly disease worldwide. In China, 110,000 people die of liver cancer annually, and account for 45% of the deaths globally. Thereinto, 70–80% of the liver cancer is related to hepatitis B [30], [31].

This study has several limitations that should be considered. First, some acute patients with hepatitis B were included in the hepatitis B carriers selected for the study. In a cross-sectional study, it is difficult to identify an acute infection due to lack of follow up, so the real prevalence of chronic hepatitis B in insular regions may be lower than that reported. Second, HBV virological assessment including HBV DNA levels and HBV genotypes were not performed. We did not analyze the clinical features of hepatitis, including jaundice, fatigue, and hepatic failure, in our community-based population, which may be significant among individuals.

In conclusion, this community-based study of insular regions provides information that might be useful in the design and implementation of future prevention strategies. Due to the geographic location, we found that HBV prevalence and potential risk of development of hepatocellular carcinoma remained high in insular regions of southeast China, and far above the national rates. Although the vaccination program has been implemented over the past 20 years, hepatitis B infection remains a great challenge for disease control in China. According to our research results, the government needs to undertake more long-term measures, such as the management of the high risk people, the floating population living on the islands, expansion of the immune population, and improved health education for fishermen.
